# Cultural practices and sexual risk behaviour among adolescent orphans and non-orphans: a qualitative study on perceptions from a community in western Kenya

**DOI:** 10.1186/1471-2458-14-84

**Published:** 2014-01-27

**Authors:** Milka Juma, Ian Askew, Jane Alaii, L Kay Bartholomew, Bart van den Borne

**Affiliations:** 1 Department of Health Promotion, Maastricht University, Maastricht, Netherlands; 2 Population Council, P.O. Box 17643, Nairobi 00500, Kenya; 3 Context Factor Solutions, P.O. Box 27598, Nairobi, Kenya; 4 University of Texas School of Public Health Houston, Houston, TX, USA

**Keywords:** Sexual risks, Cultural risk factors, Adolescents, Orphans, Non-orphans

## Abstract

**Background:**

This study explored community perceptions of cultural beliefs and practices that may increase sexual risk behaviour of adolescents, to understand more about meaning they hold within the culture and how they expose adolescent orphans and non-orphans to higher risks in a high HIV and teenage pregnancy prevalence context.

**Methods:**

Using a qualitative descriptive cross-sectional design 14 focus group discussions were conducted with 78 adolescents and 68 parents/guardians purposively selected to represent their communities. Thirteen key informant interviews were also conducted with community leaders, health care and child welfare workers, and adolescents who were also selected purposively. The two methods were used to explore how cultural beliefs and practices predispose adolescent orphans and non- orphans to risky sexual behaviours. Data were analysed through line-by-line coding, grouped into families and retrieved as themes and sub-themes.

**Results:**

Identified cultural practices that predisposed adolescents orphans and non-orphans to risky sexual behaviours included: adolescent sleeping arrangements, funeral ceremonies, replacing a deceased married daughter with her younger sister in marriage, widow inheritance among boys, early marriage among girls, and preference for boys/sons. Cultural risks perceived to equally affect both orphans and non-orphans were sleeping arrangements, funeral ceremonies, and sister replacement. Factors associated more with orphans than non-orphans were widow inheritance among boys and a preference for boy over girl children.

**Conclusions:**

Adolescent sexual risk reduction programs should be developed considering the specific cultural context, using strategies that empower communities to challenge the widely accepted cultural norms that may predispose young people in general to sexual risks while targeting those that unequally influence orphans.

## Background

Over one third (37%) of Kenyan adolescents aged 15–19 years have engaged in sex, and by age 19, 36% of the girls have given birth according to the 2008–09 Kenya Demographic and Health Survey (2008–09 KDHS) [1]. In over half of first pregnancies, the mother is unmarried, and those pregnancies are 2.4 times more likely to be reported as unintended, compared with repeat pregnancies [2]. Additionally, three percent of female adolescents in this age group are infected with HIV compared to one percent of their male counterparts in 2008–09 [1].

Of the Kenyan population aged 15–49 years, the six counties in the former Nyanza Province^a^ in western Kenya have the highest HIV prevalence rate of 14.9%, which is double the national average of 7.4% according to the 2007 Kenya AIDS Indicator Survey (2007 KAIS) [3]. The 2008–09 KDHS indicates infection rates among adolescents in this region of Kenya at 8% for female adolescents and 3% for male adolescents and the region also has the highest teenage pregnancy rate of 27% in Kenya [1].

Four of the six counties in the former Nyanza Province are located along the shores of Lake Victoria, predominantly inhabited by the Luo ethnic group. This ethnic group has the highest HIV prevalence in the country at 20%, nearly three times the national average, and bears one-third of the national HIV/AIDS burden [1,3]. The former districts of Migori, Kisumu, Homa Bay and Suba (now Migori, Kisumu and Homa Bay counties) are the focus areas for this paper. The former Suba district is now part of Homa Bay County.

The high HIV prevalence rate in the former Nyanza Province is, in part, attributable to the traditional cultural practices surrounding sexuality and gender relations of the Luo ethnic group [4-6]. An example is “widow inheritance” which is traditionally performed before a widowed woman is reintegrated into regular community activities after the husband’s death [6,7]. Widow inheritance means that the woman must marry another man from her husband’s family or village, usually a brother or close male relative of her husband. This practice exposes widows and the men to the risk of contracting or re-infection with HIV or other sexually transmitted infections (STIs) [6-9]. While effects of cultural practices such as widow inheritance on adults are known, there is lack of information on whether or not such practices affect adolescents. Cultural practices associated with elevated HIV infection and teenage pregnancy risks among adolescents in the former Nyanza Province include early marriage [10,11], sleeping arrangements and funeral ceremonies [12]. However, these studies are quantitative and provide limited understanding of how the factors predispose adolescents to sexual risks. Such high-risk cultural practices persist despite the increased awareness about HIV/AIDS among the Luo ethnic group [1].

Orphanhood has been associated with risky sexual behaviours such as early sexual debut and engaging in sex with negative health outcomes including higher incidences of HIV, STI and teenage pregnancy according to studies conducted in South Africa and Zimbabwe [13-16]. However, these studies did not explore whether or not cultural factors contribute in enhancing risky behaviour among adolescents. This study explores community perceptions of whether or not cultural factors influence the sexual behaviour and risks of adolescent orphans compared with non-orphans. Identifying the cultural factors that predispose adolescents to sexual risks and whether they affect orphans and non-orphans differently will contribute to the design of culturally responsive prevention policies and interventions for both groups of adolescents.

## Methods

### Study setting

The study was undertaken in eight sub-locations within the four former districts of Nyanza Province: Kisumu, Homa Bay, Migori and Suba. The region covers an area of 6,240 square miles (16,162 square km) with an approximate population of 5.44 million, representing 15% of the Kenyan population. The province is located in south-western Kenya along the shores of Lake Victoria and predominantly inhabited by the Luo people. The ethnic group has the highest adult (15–49) HIV prevalence of 20%, three times the national average [1]. Nyanza Province bears one-third of the national HIV/AIDS burden in Kenya [3]. The province also has the highest HIV prevalence rate among adolescents aged 15–19 at 8%, with females nearly four times as likely to be infected as their male counterparts. The area also hosts the largest proportion of orphans in Kenya at 19% [1,3]. Nyanza Province has the highest poverty incidence in Kenya with 65% of the population living below the poverty line, on less than one US dollar per day [17,18] with marked constituency level differences ranging from 43%-80%. The region contributes one-fifth of the total national poverty [17,18]. The main economic activities include subsistence farming, fishing and small-scale trade in various fields. The study areas host many fishing landing beaches that are associated with risky sexual behaviours and higher HIV prevalence rates than the average provincial rate.

Using stratified sampling of communities, two constituencies were randomly selected from each of the four districts. For each constituency, a location (an administrative unit) was randomly chosen, within which one sub-location (the smallest administrative unit) was then chosen randomly, giving a total of eight sub-locations for the study sites as shown in Table 1.

**Table 1:** Study site sampling and achieved FGDS and KIIs

**District**	**Constituency (C)**	**Location (L)**	**Sub-Location (SL) (study sites)**	**FGDs**		**KII**
				**Adolescents**	**Adults**	
District 1 Kisumu	C1.Kisumu Town West	L1.Township	SL1.Kaloleni	1	1	1
	C2.Kisumu Rural	L2.South Central Seme	SL2.West Othany	1	1	1
District 2 Migori	C3.Uriri	L3.West Kanyamkago	SL3.Kajulu1B	1	0	2
	C4.Migori	L4.Suna Ragana	SL4.Marindi	1	1	2
District 3 Homa Bay	C5.Rangwe	L5.Township	SL5.Arujo	1	1	1
	C6.Ndhiwa	L6.Kwabwai	SL6.Kachuth	1	1	2
District 4 Suba	C7.Mbita	L7.Gembe West	SL7.Kasgunga C	1	0	2
	C8.Gwassi	L8.Gwassi East	SL8.Kibwer	1	1	2
			**Total:**	**8**	**6**	**13**

A qualitative descriptive cross-sectional design was used. Focus group discussions (FGDs) and key informant interviews (KIIs) were used to get in-depth perspectives on how cultural factors predispose adolescent orphans and non-orphans (under 18 years old) to risky sexual behaviour in the eight study sites.

The first author developed the FGD and KII guides in English and translated them into the local languages, *Dholuo* and *Kiswahili*. The guides explored cultural beliefs and practices in study communities that may predispose adolescent orphans and non-orphans aged 10–17 to sexual risks and if orphans were at enhanced risks. The first author together with eight experienced qualitative researchers, pre-tested the guides in communities neighbouring the study sites for clarity of questions and to inform participants’ availability and logistics for data collection*.* The pre-test showed that adult males were less likely to be available for FGDs because of work commitments. Questions related to cultural risks are shown in Table 2.

**Table 2:** FGD and KII questions related to cultural risks

	**FGD**	**KII**
1. What are some of the common reproductive health risks faced by adolescent aged 10–17 in this community?	√	√
Probe by asking “any other?” until respondents have no additional information		
If not mentioned above, probe for teenage pregnancy and sexually transmitted and HIV infections	√	√
Do the factors differ for orphans and non-orphans?	√	√
2. a) What factors put adolescents aged 10–17 in this community at the risks mentioned above?	√	√
Probe by asking “any other?” until respondents have no additional information	√	√
For each factor mentioned, explain how it predisposes adolescent orphans and non-orphans to sexual risks	√	√
If not mentioned, probe for the following factors and explain how each may put adolescents at risk:	√	√
b) Cultural beliefs and practices,	√	√
c) Personal behaviour	√	√
d) Family socioeconomic status,	√	√
e) Community environment in general	√	√

### Consent for the study and participants’ sampling

The institutional review board of the Population Council, the Kenyatta National Hospital Ethics and Research Committee Review Board, the Ministry of Science and Technology and the national Department of Children’s Services Nairobi, approved this study. At community level, consent was obtained from the community authorities as follows: The Nyanza Provincial Children’s Officer sent copies of the letter from the national Department of Children’s Services regarding the study to the four District Children’s Officers (DCOs) who sensitized the study site community leaders (Sub-Chiefs) about the study. The first author then met with community leaders of each of the eight study sites and further briefed them on the purpose, process, and methods of data collection, what would be expected of them and sought their consent to conduct the data collection in their respective communities.

The village leaders then assisted the data collection team to identify the study areas and participants by categories. The FGD participants were selected purposively to ensure both male and female adolescents were represented equally and that male parents and caregivers of children aged 10–17 were also represented. In cognizance of the generalized HIV epidemic and poverty in these communities, we invited adolescents to participate with attention to age, sex and availability only. HIV status was not considered in the sampling to minimize the effects of stigma and to encourage free discussion on the topic. Only one person per category of adolescents and caregivers was recruited per household.

When seeking adult consent and adolescent assent, prospective participants were informed that participation in the study was voluntary, that there would be no consequence for non-participation and they could skip questions they felt uncomfortable to answer. For adolescents to participate in the study, parental consent was sought before obtaining their assent to participate. All adolescent participants had either a parent or caregiver. KII participants were assured of confidentiality when obtaining adult consent and adolescent assent and FGD participants warned to keep the discussion confidential.

### Data collection

The data collection team which included the first author and eight experienced qualitative researchers obtained informed consent from parents and caregivers themselves, adolescent assents and other adult participants using a consent form. The team was trained on key steps to obtaining consent at the household level and at the interview or FGD venue. The training included how to introduce themselves, the purpose of the study, categories of respondents and sampling procedure in cases with more than one prospective participants per category at the household. The researchers then sought verbal consent from those identified to participate and informed them to meet at a central place in the community on a specific date. Each participant also gave consent at the data collection level.

Fourteen FGDs were conducted with 147 participants; eight with 78 adolescents ages 14–17 (four with boys and four with girls) and six with 69 caregivers in two age groups of 25–49 years (i.e. those in the reproductive age group) and 50 years and above (older caregivers), distributed by gender. Of the six caregiver FGDs, four were with females and two with males; each FGD had 10–12 participants. Focus group discussions and KIIs were conducted in *Kiswahili*, *Dholuo* or English depending on respondents’ language preference; they were audio-taped with participants’ consent and lasted between one to two hours.

Thirteen key informants were also purposively selected because they were knowledgeable of the study communities and either lived in or worked in or with the communities on issues affecting the members, orphaned families and adolescents. They 13 key informants comprised: five community leaders; three child welfare workers including a District Children’s Officer (DCO) and two Area Advisory Council (AACs) representatives, three health care workers and a male and a female adolescent. The data collection ended when no new information emerged.

### Data analysis

The audio-taped FGDs and KIIs were transcribed verbatim, translated into English when needed and typed as Microsoft Word documents. The first author read all FGDs and KIIs, and developed an initial coding scheme that was reviewed and refined by the fourth and fifth authors. ATLAS.ti 5.2 software was used for line-by-line coding and grouping the initial codes into themes of emerging cultural factors associated with sexual risks among adolescents [19,20].

## Results

### Background characteristics

Of the 147 FGD participants, 53% were adolescents and 47% were caregivers. The adolescent participants comprised of 47% and 53% females and males respectively, while among caregivers 65% and 35% were females and males respectively. Detailed characteristics of adolescents, caregivers and key informants are shown in Table 3.

**Table 3:** Demographic characteristics of participants

**Characteristics**	**Study categories by data collection methods**		
	**FGD**		**KII**
	**Parents/caregivers**	**Adolescents**	
	**(n = 69)**	**(n = 78)**	**(n = 13)**
**Sex**			
Male	24 (35%)	37 (47%)	9 (69%)
Female	45 (65%)	41 (53%)	4 (31%)
**Age group**			
14–17	-	78 (100%)	2 (15%)
30–49	33 (48%)	-	7 (54%)
50–72	36 (52%)	-	4 (51%)
**Highest level of education**			
No education	11 (16%)	-	-
Primary	27 (39%)	48 (62%)	2 (15%)
Secondary	25 (36%)	30 (38%)	5 (39%)
Post-Secondary	6 (9%)	-	6 (46%)
**Marital Status**			
Single	-	78 (100%)	2 (15%)
Married	42 (61%)	-	11 (85%)
Widowed	27 (39%)	-	-
**Categories of key**			
**Informants**			
Adolescents	-	-	2 (15%)
Community leaders	-	-	5 (39%)
Child welfare workers	-	-	3 (23%)
Health care providers (HCP)	-	-	3 (23%)

### Cultural themes

Six cultural themes emerged from the data analysis: adolescent sleeping arrangements, funeral ceremonies, replacement in marriage of a deceased married daughter by her younger sister, widow inheritance, early marriage and boy-child preference. Table 4 shows the themes and the number of times each is mentioned by data collection method. Data describing these themes are aggregated across sources and the sources are indicated to facilitate interpretation.

**Table 4:** Cultural themes by number and percentage of FGDs and KIIs

	**Adol. FGDs**	**Adult FGDs**	**KII**
	**n = 8**	**n = 6**	**n = 13**
	**n (%)**	**n (%)**	**n (%)**
Sleeping arrangements	5 (63%)	2 (33%)	5 (39%)
Funeral ceremonies	5 (63%)	3 (50%)	6 (39%)
Sister replacement in marriage	4 (50%)	2 (33%)	3 (23%)
Widow inheritance	4 (50%)	3 (50%)	4 (31%)
Early marriage	4 (50%)	3 (50%)	6 (39%)
Boy-child preference	4 (50%)	2 (33%)	3 (23%)

### Sleeping arrangements

Adolescents and male caregivers frequently reported the Luo cultural tradition that prohibits children who have reached puberty from sleeping in the same house as their parents. Traditionally, adolescents either slept at their grandparents’ house, in the household kitchen (usually a separate structure), at a brother’s house within or outside the homestead, or at a neighbour’s house. Female adolescents and male caregivers suggested that such sleeping arrangements provided opportunities for adolescent orphans and non-orphans to sneak out at night to attend discos and to meet with girlfriends/boyfriends, contexts that predisposed them to risky sexual activity, either willingly or through force.

“At the neighbour’s, there is a lot of influence to have sex because there is freedom of interactions. Sometimes the neighbour’s compound may be distant, so as the girl walks there at night, a boy can intercept and rape or sweet-talk her into sex.” (Male caregiver, FGD)

“Boys who sleep in their brothers’ houses within their homesteads sometimes sneak in their girlfriends for sex at night. I also know that girls who sleep in the grandmothers’ houses easily go for night dances because the grandmother cannot stop them. At the dance, a lot of sex talk goes on between boys and girls and it may be hard to resist some of these temptations.” (Female adolescent, FGD)

### Funeral ceremonies

The frequency of AIDS-related deaths in the study communities and the elaborate funeral ceremonies that are often held over several days were reported by adolescents, caregivers, community leaders and child welfare workers to be conducive environments for adolescents to engage in casual sex. During funeral ceremonies large numbers of community members, including orphaned and non-orphaned children, congregate for long periods at the home of the deceased where there are overnight prayers, music and dancing (“funeral discos”).

Orphaned and non-orphaned adolescents are not often monitored closely by caregivers and so have opportunities for engaging in sex.

“…we have funeral festivities where in this community there is music at night. Funeral activities take place for many days before and after the burial. During these occasions, you find the adolescents are the ones participating in the dances especially those who are not monitored keenly. Boys meet girls whom they lure into casual sex that is usually unprotected. I doubt that they use condoms.” (AAC representative, KII)

### Replacement of a deceased married daughter with her younger sister

The Luo cultural practice of replacing a deceased married daughter with a younger sister was identified as a sexual risk in discussions with caregivers, adolescents adult men and women, adolescent girls and boys and in three key informant interviews:

“It is the social responsibility on the parents of a married deceased daughter to get the widowed son-in-law another wife. If there is no mature daughter and there is a younger one, she’ll be married off to replace her late sister and it is acceptable in this community.” (DCO, KII).

Caregivers revealed two cultural values of sister replacement: ensures continuity of the relationship between in-laws and care of the deceased woman’s children. However, some study participants, particularly female caregivers and girls, stated that this practice puts young girls at risk of unprotected sex, probably with a HIV-infected partner if the sister died of AIDS or the in-law is infected, thereby exposing them to a range of reproductive health risks.

“Sister replacement often results in early marriage and the risks of early birth and HIV infection from her deceased sister’s husband.” (Female caregiver, FGD)

The practice of sister replacement forces girls to marry at a young age as a cultural obligation, sometimes to a man whose age is more than double her age, and in some cases against their will.

“When a married woman dies, the younger unmarried sister is 'forced’ to replace her despite her young age. Further, when your sister is ailing, she may call you and ask you to come and care for her children (marry her husband) when she dies. You will have to honour her request even if you are not sure of what she died from.” (Female adolescent, FGD)

Discussions with adolescents and caregivers on vulnerability of orphaned girls versus non- orphans to sister replacement, indicated that both categories were at risk and the decision depended on the girl or her caregivers. Adolescents and caregivers also reported household poverty as a factor that influenced both orphan and non-orphan adolescents to accept sister replacement marriage.

“Both orphans and non-orphans are affected by this cultural practice of sister replacement as it often decided by parents or caregivers but sometimes the girls are also willing especially when the man is wealthy.” (Male caregiver, FGD)

### Widow inheritance

Widow inheritance was reported by caregivers, adolescents and key informants as a common cultural practice among the Luo ethnic group that can sometimes put male adolescents at risk of HIV/STI infection. Adolescent and caregiver respondents reported that the cultural practice requires a widow be inherited by an immediate brother-in-law or other appropriate male relative of her deceased husband. In the absence of such a relative, male adolescents from one study site reported that some families prevail upon their male adolescent sons to inherit their widowed in-laws to keep off men from outside the family. Male adolescents from the specific study community felt obliged to engage in widow inheritance.

“You can be told that you are the one to inherit your brother’s wife and when you are told by the elders, you cannot refuse even if you are still in school. You may not know what killed your brother so you just inherit the woman as a result of this practice which can lead to infection with diseases”. (Male adolescent, FGD)

Male adults and adolescents reported that boys, especially orphans from poor households, to be more vulnerable to engaging in widow inheritance, particularly those who are hired as cattle herders or farm workers in communities other than theirs.

“Poverty in some households, especially orphaned households may force a boy in such a household to become a herdsman in a different community. If he finds better diet there, he opts to stay at that home. Such boys end up inheriting women whose husbands have died without knowing what killed the husband. This is how young boys get infected with STI/HIV and die.” (Male caregiver, FGD)

### Early marriage

Early marriage was discussed by female adolescents, caregivers, and in interviews with child welfare and health care workers and a male adolescent. Girls reported that some parents encouraged early marriage to get cattle for dowry, resulting in school dropout. This was perceived to put girls at risk for STI including HIV infection.

Early marriage is inherent in this community. I handle many cases of early marriage in my office here. The community accepts and is silent about it”. (DCO, KII)

“There are parents who discourage their daughters from going to school. They encourage them to get married so that the prospective husbands pay dowry by bringing cows that the mother can milk and 'enjoy life’. Such a parent will not focus on educating the daughter even if this child would like to proceed with her education”. (Female adolescent, FGD)

### Boy preference

Adolescents, caregivers and key informants reported boys as culturally valued over girls, thus resulting in preferential treatment of boys by caregivers. This is especially so among resource constrained families, for which boys are perceived to be potentially important future sources of support. Conversely girls are perceived to belong elsewhere as they will be married in other communities and may not be beneficial to their parents or caregivers in future. Orphan girls living in poor households were reported to be at increased risk of such discrimination. This norm reportedly makes girls feel less valued and may discourage them from continuing to live in their homes, with some choosing marriage and some engaging in risky sexual behaviours to feel valued. Female participants in all categories mentioned preferential treatment of boys as a contributing factor to early marriage.

“Some parents follow the Luo traditional belief that girls are *“ogwenge”* (“wild cats”) who will be married elsewhere. Many parents bank on boys for future assistance than girls. Girls are often taken for granted. This is one source of problem that makes girls walk out of their homes easily and get into these risks.” (Male caregiver, FGD)

“In this community boys are more valued than girls. If a brother and a sister pass exams at the same time and the family has limited resources, the boy will proceed to secondary school while the girl will be left at home. Among orphans, I have observed instances where the male orphan goes for secondary education while the sister with equally good grades is left at home where guardians’ resources are scarce.” (Female adolescent, KII)

## Discussion

Our study identified a range of cultural practices that exposed orphan and non-orphan adolescents (10–17 years) to sexual risks. These included: adolescents sleeping in a separate house from parents or caregivers, attending funeral ceremonies during the night, young girls replacing to a deceased older sister in marriage, widow inheritance by an immediate brother-in-law or other male relative of her deceased husband, early marriage and preference for boys. Cultural risks that seemed to affect orphans more than non-orphans included boys engaging in widow inheritance and preference for boys to girls resulting in discrimination against girls, when resources are scarce with boys being given preference, for example in education.

Traditionally, adolescents slept in a separate house to allowed parents the privacy for intimacy. The sleeping arrangements also played an important role in socialization of adolescents on sexuality and moral values by grandparents and older relatives usually along gender lines. The socialization inculcated values that helped the adolescents avoid risky behaviours as they grew [12]. However, with the breakdown of traditional social fabrics, such structures no longer exist leaving a gap in sources of sexuality information for adolescents [12,21-24]. The adolescents’ sleeping arrangements now leave them unsupervised and with the opportunity to sneak out at night to attend discos and other social activities where they have the freedom to engage in casual sex that is often unprotected [12].

Traditionally, music and dance have been an integral part of *Luo* funerals [12,25]. This study found funeral ceremonies, especially the night dances/discos and overnight prayer gatherings, to create environments conducive for risky sexual behaviour. This is consistent with results from other studies in the same locality [12,26]. However, funeral ceremonies can be used as avenues for prevention education and services, not only for adolescents but also adults and caregivers in general.

Our study suggests that sister replacement in marriage often contributes to early marriage, a practice that is associated with early pregnancy, sexual violence and STI and HIV risks [27-30], partly attributable to the large age differences between the girls and their male spouses [31-34]. However, while sister replacement helps with continuity of the relationship between the in-laws and care of the orphaned children, the *Luo* community needs to review these values versus the risks posed to the girl and embrace prevention strategies that reduce risky behaviour. Therefore prevention interventions should include sensitising communities on risks of sister replacement and identifying appropriate people to train as awareness and behaviour change promoters.

In this study, poverty was found to motivate early marriage, consistent with results from a study on facilitators of sexual risk behaviours among orphans and non-orphans in the same study area [12,35] and also with a report on causes and consequences of early marriage. [36]. Poverty also exacerbated sister replacement, poor boys inheriting widows in communities where they work to earn a living and boy preference. The risks of widow inheritance among male adolescents, and the preferential treatment of boys over girls, were more associated with orphans. This suggests that households caring for orphans may be more resource constrained than those without orphans [37,38]; thus, poverty is likely to perpetuate negative cultural practices and should be addressed as part of the response to cultural risks. Our results indicate a need to implement appropriate economic empowerment interventions to discourage such cultural practices and enable families to provide for their basic needs.

Our qualitative study has some strengths and limitations. Study respondents participated voluntarily having been assured that there would be no consequence for non-participation. Additionally, orphanhood was not used as a criteria for selecting adolescent or parents/caregivers participants to avoid any stigma. The study region has a generalised HIV epidemic and therefore those affected by HIV deaths would be represented. Further participants discussed sexual risk factors in general and not their personal experiences. The three approaches allowed FGD participants to discuss freely without fear of stigma.

Study participants were asked to mention cultural factors that predispose adolescent orphans and non-orphans to sexual risks. Culture is a way of life and it is possible that some respondents may not have recognized the negative effects of certain cultural practices. Our data may not be representative of the adolescent orphans and non-orphans in the former Nyanza Province. However, the results provide insights into cultural practices that can inform reproductive health and HIV risk prevention interventions. Further qualitative and quantitative research on the identified cultural risks is needed to gain a deeper understanding of the issues and to explore other risks that may not have been mentioned by participants. Such a study should also take account of married adolescents, including those married in the context of sister replacement, to gain an insight into community views on how interventions could address cultural risks while honouring the positive aspects of the traditional practices.

## Conclusion

Cultural practices, including adolescent sleeping arrangements, night funeral ceremonies, replacement of a deceased sister in marriage, widow inheritance, early marriage, and preference for boys contribute to risky sexual behaviours that predispose adolescent orphans and non-orphans to teenage pregnancy and STI/HIV infection. Poverty exacerbates risky cultural practices; thus prevention interventions should target poverty as well as risky cultural factors such as adolescents’ sleeping arrangements, participation in night funeral gatherings, sister replacement, early marriage and preferential treatment of boys over girls. In that way, we may reduce and possibly eliminate their risks to adolescent orphans and non-orphans.

### Endnote

^a^The former eight provinces of Kenya have been changed to 47 counties according to the 2010 Constitution of Kenya. The former Nyanza Province has six administrative counties namely: Kisii, Nyamira, Siaya, Kisumu, Migori and Homa Bay.

## Abbreviations

AAC: Area advisory council; DCO: District children’s officer; FGD: Focus group discussion; KII: Key informant interview; STI: Sexually transmitted infection.

## Competing interest

The authors declare that they have no competing interests.

## Authors’ contributions

MJ and IA equally made substantial contributions to the conception and design of the study. MJ also drafted data collection instruments, trained data collectors, participated in pre-test, data collection, analysis and interpretation, drafted this paper, and was responsible for submission. IA and JA also edited the draft versions of the data collection instruments, and edited the various drafts of this paper. LKB made substantial contributions to data analysis and interpretation, and revised the first draft of the paper and version to be submitted. IA, and. BVB made substantial contributions to the data analysis, interpretation, edited draft versions of this manuscript and the version to be submitted. All authors read and approved the final manuscript.

## Pre-publication history

The pre-publication history for this paper can be accessed here:

http://www.biomedcentral.com/1471-2458/14/84/prepub

## References

[B1] Kenya National Bureau of Statistics and ICF Macro Kenya Demographic and Health Survey 2008–09 2010 Calverton, Maryland: KNBS and ICF Macro

[B2] Taffa N Omollo D Mathewa Z Teenage pregnancy experience in Kenya Int J Adolesc Med 2003 15 4 331 340 10.1515/ijamh.2003.15.4.331 14719415

[B3] National AIDS and STI Control Programme Kenya AIDS Indicator Survey, KAIS 2007, Final Report 2009 Nairobi, Kenya: Ministry of Health

[B4] Oluoch CA Nyongesa WJ Perceptions of the Luo community on widow inheritance and HIV/AIDS in Kenya: towards developing risk communication messages Int J Bus Soc Sci 2013 4 1 213 219

[B5] Kawango EA Stoep AV Tracy M Obare BA Bukusi EA Widow inheritance and HIV prevalence in Bondo District, Kenya: Baseline results from a prospective cohort study PloS One 2010 5 11 e14028 doi:10.1371/journal.pone.0014028 10.1371/journal.pone.0014028 21103347 PMC2984493

[B6] Institute of Policy Analysis & Research HIV/AIDS Scourge in Nyanza Province: poverty, culture and behavior change Policy Brief 2004 10 11 1 4

[B7] Ambasa-Shisanya CR Widowhood in the era of HIV/AIDS: a case study of Siaya District, Kenya SAHARA J 2007 4 2 606 615 10.1080/17290376.2007.9724882 18071612

[B8] Ocholla-Ayayo ABC Psychical, Social and Cultural Issues Relating to HIV/AIDS Containment and Transmission in Africa with Special Reference to Kenya; Population Studies Research Institute 1996 Nairobi: University of Nairobi

[B9] Agot BO Agot K Agingu WO Bukusi EA Recognizing and Addressing Tensions Between Culture and HIV Prevention: Lessons from a Study on Widow Inheritance 2004 Bangkok, Thailand: 15th International Conference on AIDS

[B10] Erulkar A Ayuka F Addressing Early Marriage in Areas of High HIV Prevalence: A program to Delay Marriage and Support Married Girls in Rural Nyanza, Kenya Promoting healthy, Safe, and Productive transition to adulthood 2007 Population Council: Frontiers in Reproductive Health, Brief No. 19 7

[B11] Glynn JR Carael M Auvert B Kahindo M Chege J Musonda R Kaona F Buve A Why do young women have a much higher prevalence of HIV than young men? A study in Kisumu, Kenya and Ndola, Zambia AIDS 2001 15 suppl 4 S51 S60 11686466 10.1097/00002030-200108004-00006

[B12] Juma M Alaii J Bartholomew LK Askew I Borne BV Risky sexual behavior among orphan and non-orphan adolescents in Nyanza Province, western Kenya AIDS Behav 2012 DOI: 10.1007/s10461-012-0336-5 10.1007/s10461-012-0336-5 23073645

[B13] Thurman TR Brown L Richter L Sexual risk behavior among South African adolescents: Is orphan status a factor? AIDS Behav 2006 10 6 627 635 10.1007/s10461-006-9104-8 16838071

[B14] Hallman K “Orphanhood and Adolescent HIV Risk Behaviors in KwaZulu-Natal, South Africa” 2006 Los Angeles, California: Paper Presented at the Population Association of America 2006 Annual Meeting

[B15] Gregson S Nyamukapa CA Garnett GP HIV infection and reproductive health in teenage women orphaned and made vulnerable by AIDS in Zimbabwe AIDS Care 2005 17 7 785 794 10.1080/09540120500258029 16120495

[B16] Birdthistle IJ Floyd S Machingura A Mudziwapasi N Gregson S Glynn JR From affected to infected? Orphanhood and HIV risk among female adolescents in urban Zimbabwe AIDS 2008 22 6 759 766 10.1097/QAD.0b013e3282f4cac7 18356606

[B17] Central Bureau of Statistics Geographic dimensions of well-being in Kenya. who and where are the poor? a constituency level profile 2005 Government Printers: Volume II. Nairobi

[B18] Kenya National Bureau of Statistics Basic report on well-being in Kenya. Based on the Kenya integrated household budget survey 2005/06 2007 Nairobi: Ministry of Planning and National Development

[B19] Muhr T ATLAS.ti 5.2 Scientific Software Development 2006 Berlin, Germany: GmbH

[B20] Charmaz K Constructing Grounded Theory: A Practical Guide Through Qualitative Analysis 2006 London/ Thousand Oaks/ New Delhi: SAGE Publications Ltd

[B21] Mbugua N Factors inhibiting educated mothers in Kenya from giving meaningful sex-education to their daughters Soc Sci Med 2007 64 1079 1089.20 10.1016/j.socscimed.2006.10.008 17258368

[B22] Odek T Cultural Challenges and Sex Education in Mageta Island, Kenya. Post- Sexuality Leadership Development Fellowship Report Series No. 2 2007 Africa Regional Sexuality Resource Centre

[B23] Kayongo-Male and Onyango The Sociology of the African Family 1984 Kew York: Longman group Ltd

[B24] Jensen DF Growing up Sexually, Vol.1, World Reference Atlas, 0.2 (ed.) 2005 Berlin: Magnus Hirschfield for sexology

[B25] Mboya P Luo Kitgi gi Timbegi. Sixth Printing 1997 Kisumu, Kenya: Anyange Press 176

[B26] Njue C Voeten HA Remes P Disco Funeral: a risk situation for HIV infection among youth in Kisumu AIDS 2009 23 4 505 9 10.1097/QAD.0b013e32832605d0 19165086 PMC2675523

[B27] Huebner AJ Howell LW Examining the relationship between adolescent sexual risk- taking and perceptions of monitoring, communication, and parenting styles J Adolesc Health 2003 33 2 71 78 12890597 10.1016/s1054-139x(03)00141-1

[B28] Babalola S Tambashe BO Vondrasek C Parental factors and sexual risk-taking among young people in Cote d’Ivoire Afr J Reprod Health 2005 9 1 49 65 10.2307/3583160 16104655

[B29] Mavedzenge SMN Doyle AM Ross DA HIV prevention in young people in Sub Saharan Africa: a systematic review J Adolesc Health 2011 49 6 568 586 10.1016/j.jadohealth.2011.02.007 22098767

[B30] Bearinger LH Sieving RE Ferguson J Sharma V Global perspectives on the sexual and reproductive health of adolescents: patterns, prevention, and potential Lancet 2007 369 9568 1220 1231 10.1016/S0140-6736(07)60367-5 17416266

[B31] Clark S Early marriage and HIV risks in Sub-Saharan Africa Stud Fam Plann 2004 35 3 149 160 10.1111/j.1728-4465.2004.00019.x 15511059

[B32] Clark S Bruce J Dude A Protecting young women from HIV/AIDS: The case against child and adolescent marriage Int Fam Plan Perspect 2006 32 2 79 88 10.1363/3207906 16837388

[B33] Kelly RJ Gray RH Sewankambo NK Serwadda D Wabwire-Mangen F Lutalo T Wawer MJ Age differences in sexual partners and risk of HIV-1 infection in rural Uganda JAIDS 2003 32 445 51 10.1097/00126334-200304010-00016 12640205

[B34] International Women’s Health Coalition Triple Jeopardy: Female Adolescence, Sexual Violence, and HIV/AIDS 2008 New York: IWHC

[B35] Juma M Alaii J Bartholomew LK Askew I Borne van den B Understanding orphan and non-orphan adolescents’ sexual risks in the context of poverty: a qualitative study in Nyanza Province, Kenya BMC Int Health Hum Rights 2013 13 32 doi: 10.1186/1472-698X-13-32 10.1186/1472-698X-13-32 23886019 PMC3725178

[B36] Pathfinder International Report on Causes and Consequences of Early Marriage in Amhara Region 2006 Addis Ababa: Pathfinder International

[B37] Abebe T Orphanhood, Poverty and Care Dilemma 2009 Norway: Review of Global Policy Trend

[B38] Ssewamala FM Chang-Keun H Torsten B Effect of Economic Assets on Sexual Risk-Taking Intentions among Orphaned Adolescents in Uganda AM J Public Health 2010 100 3 483 488 10.2105/AJPH.2008.158840 20075323 PMC2820050

